# Problematic short drama addiction among Chinese Gen X women: a ZMET approach

**DOI:** 10.3389/fpsyg.2026.1862137

**Published:** 2026-07-08

**Authors:** Yuelin Tan

**Affiliations:** School of Business, Ludong University, Yantai, China

**Keywords:** addictive behavior, consensus map, Gen X women, short drama addiction, ZMET

## Abstract

**Background:**

Short drama addiction has emerged as a novel form of behavioral addiction in the digital age. Chinese Gen X women (born 1965–1980), the core user and high-risk group for this addiction, have had the underlying mechanisms of their addictive behavior not yet elucidated. Existing research has critical gaps in exploring the subconscious drivers of short drama addiction in this specific generational-gender population, alongside methodological limitations of traditional quantitative approaches.

**Methods:**

This qualitative study used the Zaltman Metaphor Elicitation Technique (ZMET) combined with semi-structured in-depth interviews. 36 participants with typical short drama addiction behaviors were recruited via snowball sampling and screened using the Revised Personal Involvement Inventory (RPII) scale. Following the 10 steps of ZMET, we performed data analysis through hierarchical construct extraction, personal mental mapping, and group consensus map construction.

**Results:**

71 constructs regarding short drama addiction were extracted, a group consensus map of addiction in this cohort was built, and four core consensus themes were identified, which reconstructed the dynamic evolution of their addictive behaviors.

**Conclusion:**

This study concludes that short drama addiction among Chinese Gen X women is primarily driven by emotional stress and psychological compensation needs, reinforced by poor time and attention management, leading to significant physical and mental health impacts, while also prompting growing awareness and efforts toward more rational and controlled media consumption.

## 1 Introduction

The evolution of digital media technology and the advancement of the content industry have positioned short dramas as a prominent form of media consumption in China. Their rapid growth and significant social impact have transformed patterns of mass entertainment (Chen et al., 2023). Characterized by fragmented episodes lasting 5 to 15 min and intense plot conflicts, short dramas resonate with the fast-paced lifestyle of contemporary society. They swiftly engage a wide range of age and gender demographics, emerging as the most influential digital entertainment medium following short videos (Feng, 2025). As of June 2025, the number of short drama users in China reached 626 million, accounting for 55.8% of the total internet user base. Among these users, Generation X women (born between 1965 and 1980) comprise nearly 40%, with a daily viewing duration surpassing 120 min. Their addictive behaviors, such as immersive viewing and the purchase of full-series unlocks, are notably more prevalent than those observed in other age groups (China Internet Network Information Center, 2025; China Netcasting Services Association, 2025). Short drama addiction among Gen X women reflects the intersection of traditional family roles and professional social identities. It signifies not only alienated digital media consumption but also encapsulates the emotional anxiety, role conflict, and psychological compensation needs prevalent among middle-aged women amidst social transformation (Li, L. et al., 2025; Li, W. et al., 2025). From an academic standpoint, investigating the addiction motives of this group addresses a theoretical gap in the subfield of media addiction research that concentrates on specific generational and gendered populations. This examination also offers empirical support for understanding the logic of digital media practices and the pathways of identity construction among contemporary middle-aged women. From a practical standpoint, this research provides a valuable decision-making framework for promoting healthy digital media consumption, enhancing the content ecology of short dramas, and addressing the mental health needs of middle-aged women. Consequently, it possesses significant applied research value.

Previous research has shown that women may use digital media as a means of emotional regulation and stress relief, especially under conditions of chronic emotional tension or insufficient social support (Chowdhury et al., 2025). However, existing studies have largely focused on adolescents, college students, or generalized populations of “middle-aged users” with limited attention to the specific experiences of Chinese Gen X women. Furthermore, prior studies have predominantly employed quantitative approaches emphasizing explicit motivations and behavioral frequency. These implicit factors are often challenging to assess through conventional questionnaire surveys. The Zaltman Metaphor Elicitation Technique (ZMET), a qualitative method developed to uncover participants’ deep-seated psychological metaphors, has been widely applied in marketing, sociology, and related fields (Khoo-Lattimore and Prideaux, 2013). By integrating image-based elicitation with textual analysis, ZMET is capable of capturing more than 95% of participants’ opinions once data saturation is achieved (Zaltman and Coulter, 1995). This approach enables researchers to gain deeper insights into participants’ thoughts, explore underlying metaphorical meanings, and identify previously overlooked dimensions of the research topic. This offers a viable methodological approach for elucidating the underlying mechanisms of short drama addiction among Gen X women. It addresses existing research gaps concerning specific populations, implicit driving factors, and methodological constraints.

Despite the relatively late emergence of short dramas, research on new media addiction among internet generations has accumulated a substantial theoretical foundation (Young, 1998). Some scholars have redefined the construct of “internet addiction,” arguing that the internet itself is not an addictive object, but rather a medium that may facilitate addiction to specific behaviors. The umbrella term “internet addiction” should be replaced by behavior-specific nomenclature, rather than being used as a generalized label (Starcevic and Aboujaoude, 2017). This reconceptualization has led to the emergence of targeted addiction concepts, including the present “short drama addiction.” With the rise of short video platforms, specific theories such as attachment theory have also been applied to explore the issue of short video addiction (Zhang et al., 2019). In recent years, research has been further refined to examine usage motivations and underlying psychological processes (Huang et al., 2022). In the Chinese context, scholars have examined the antecedents, cultural roots, psychological mechanisms, and coping strategies associated with short video addiction (Liao, 2024). They have developed a multidimensional explanatory framework that encompasses individual, social environment, and platform factors (Xiong et al., 2024). Regarding population-specific research, college students, older adults, and young women have been examined as target groups to explore the antecedents and influencing factors of short video or short drama addiction (Li, L. et al., 2025; Li, W. et al., 2025; Zhan and Zhu, 2025; Sun and Wu, 2025). Overall, existing research on short video and short drama addiction has evolved from identifying influencing factors to exploring underlying mechanisms, content effects, and population-specific heterogeneity. Nonetheless, a significant gap persists in empirical research regarding the underlying drivers of short drama addiction among Chinese Gen X women. Additionally, there is a deficiency of qualitative studies that investigate the subconscious psychological needs of this demographic. This study seeks to address these gaps.

Against this backdrop, the present study makes two key innovations. First, from a research perspective, we redirect the analytical focus from “behavioral addiction” to “emotional immersion and symbolic interpretation.” This approach situates the addictive behaviors of Gen X women within an integrated framework that encompasses gender roles, generational differences, and social context, allowing for an analysis of the dynamic interaction between media content and group psychology. Second, regarding methodology, we confront the limitations of quantitative research and superficial text analysis by employing a qualitative approach aimed at exploring audiences’ subconscious psychology and metaphorical cognition. This approach captures profound motivations that cannot be directly expressed through language. As a qualitative method, ZMET facilitates participants in articulating their internal cognition through image metaphors. The resulting consensus map effectively reveals subconscious emotions and attitudes (Coulter et al., 2001), which aligns with the current study’s objective of uncovering the underlying psychological mechanisms of short drama addiction. Through these innovations, this study explores the implicit psychological mechanisms of short drama addiction among Gen X women, providing new theoretical perspectives and methodological approaches for related research.

This study employs ZMET combined with semi-structured in-depth interviews, focusing on Chinese Gen X women. Using snowball sampling, we initially recruited 40 potential participants, with 36 meeting the inclusion criteria for final analysis. We implemented a structured protocol including storytelling, image classification, personal mental mapping, and consensus mapping to systematically uncover the underlying motivations and metaphors driving short drama addiction in this group. The study addresses two research questions: (1) What are participants’ attitudes and perceptions toward short drama addiction? (2) What are the underlying antecedents and formation processes of short drama addiction in this group? By focusing on subjective experiences rather than clinical diagnosis, this study aims to contribute to the literature on digital media use, gender, and emotional life in contemporary China while also demonstrating the value of qualitative metaphor-based approaches in media research.

## 2 Materials and methods

### 2.1 Study participants

Currently, there is no unified definition of short drama addiction in academia. In the Chinese context, “short video addiction” and “short drama addiction” originate from “internet addiction,” referring to dependence on the Internet, not a clinically diagnosed condition (Southern Metropolis Daily, 2009). Official Chinese documents define it as “impulsive internet use behavior caused by non-substance intake” (China Health Education Center, 2018). Therefore, the “addiction” in this study does not refer to drug addiction in clinical medicine, but rather a neutral description of the behavior leading to dependence on short dramas. This study referenced criteria for “short video addiction” and “internet addiction” from previous research when selecting participants (Feng et al., 2024; Zhang et al., 2023). Eligible participants had to meet two inclusion criteria: (1) female, born between 1965 and 1980 (Gen X); (2) exhibiting symptoms of short drama addiction, characterized by a daily viewing duration of at least 4 h, with continuous viewing sessions lasting no less than 30 min, and accompanied by an inability to curb viewing behavior despite an awareness of negative consequences. This definition aligns with widely recognized behavioral addiction criteria in academic research (Baumeister, 2014).

The initial cohort of participants was recruited via the researchers’ personal social networks (relatives, friends, colleagues) and online channels, to facilitate rapport and trust-building with participants. Snowball sampling was then implemented, with enrolled participants asked to recommend other eligible individuals willing to participate in the study, resulting in an initial sample of 40 participants. All study procedures involving human participants complied with the ethical principles of relevant Chinese regulations. Before the interview, the full study protocol was explained to all participants, who provided informed consent and were assured that all data would be presented anonymously. To improve transparency, additional sociodemographic information was collected during the interviews, including participants’ employment status, marital status, caregiving responsibilities, and educational background. Participants included retired individuals, full-time employees, self-employed workers, and full-time caregivers, reflecting diverse life situations among Chinese Gen X women.

### 2.2 Involvement test

To ensure participants had sufficient understanding of the research topic and that their narratives reflected genuine views, we administered an involvement test prior to implementing the ZMET. The test was conducted in January 2026 using the Revised Personal Involvement Inventory (RPII) scale, a validated tool to assess participants’ engagement with the research topic (Zaichkowsky, 1994). While the RPII is not a tool designed to diagnose problem use or addiction, it ensures participants’ understanding of the research topic. Therefore, the purpose of using the RPII is to guarantee each participant’s familiarity with the short drama. The scale consists of 10 items rated on a 7-point Likert scale, with a total possible score of 70. A score above 50 was defined as high involvement, indicating participants had a clear understanding of the research topic. The scale was adapted to the study context, with all items framed around the prompt “Short dramas are for you?.” To reduce random response bias, reverse-coded items were evenly distributed across the scale (Table 1). Of the initial 40 participants, 36 achieved a score above 50 (*M* = 54), indicating high involvement. These 36 participants were retained as the final study sample, as their data were deemed to reflect genuine and informed opinions.

**Table 1:** RPII scale for participants’ involvement test.

Short dramas for you?			
No.	Descriptions	Score	Descriptions
1	Important	7 6 5 4 3 2 1	Unimportant*
2	Uninterested	1 2 3 4 5 6 7	Interested*
3	Relevant	7 6 5 4 3 2 1	Irrelevant*
4	Unexcited	1 2 3 4 5 6 7	Excited*
5	Unsignificant	1 2 3 4 5 6 7	Significant*
6	Appealing	7 6 5 4 3 2 1	Unappealing*
7	Desirable	7 6 5 4 3 2 1	Undesirable*
8	Worthless	1 2 3 4 5 6 7	Valuable*
9	Beneficial	7 6 5 4 3 2 1	Not beneficial*
10	Not needed	1 2 3 4 5 6 7	Needed*

*Can be considered as the antonym of the word to its left.

Of the 36 participants, 23 were from eastern China, 8 from central China, and 5 from western China. Due to the geographical distribution of these participants, 19 were surveyed via video interviews for both the RPII test and the ZMET. Regarding education level, 8 had a bachelor’s degree or higher; 14 had a high school diploma; 11 had a junior high school diploma; and 3 had a primary school diploma. Four of the 36 participants were unemployed, yet they received financial support from their spouses or children. Seventeen were retired (the current average age for most Chinese women is 50), nine worked in private companies, and six worked in state-owned enterprises. The average income of these participants with pensions and employment was RMB 5,318. Regarding marital status, all participants were married except for three who were divorced. Furthermore, 16 participants had grandchildren and needed to help their working children care for them.

### 2.3 ZMET data collection preparation

Before the formal data collection, all researchers underwent standardized training in ZMET to ensure a comprehensive understanding of each step in the process. They practiced maintaining natural, non-directive interactions with participants to foster a relaxed interview environment that encourages full and authentic expression of insights. Using interview data, researchers identified participants’ fundamental perceptual elements and constructs, subsequently integrating these into a consensus map that reflects shared characteristics of individual mental maps. This approach visually represents the collective cognition of participants regarding the research topic. Seven to ten days prior to the formal interview, participants received a briefing on the ZMET protocol and its requirements. They were instructed to prepare 8 to 12 images that accurately represented their cognitive and emotional experiences related to short drama addiction, following a thorough reflection on the research topic. Eligible images included original photos taken by participants via mobile phones or cameras, as well as images collected from the internet, books, or other channels that met the study requirements.

### 2.4 ZMET began

**Figure 1:**
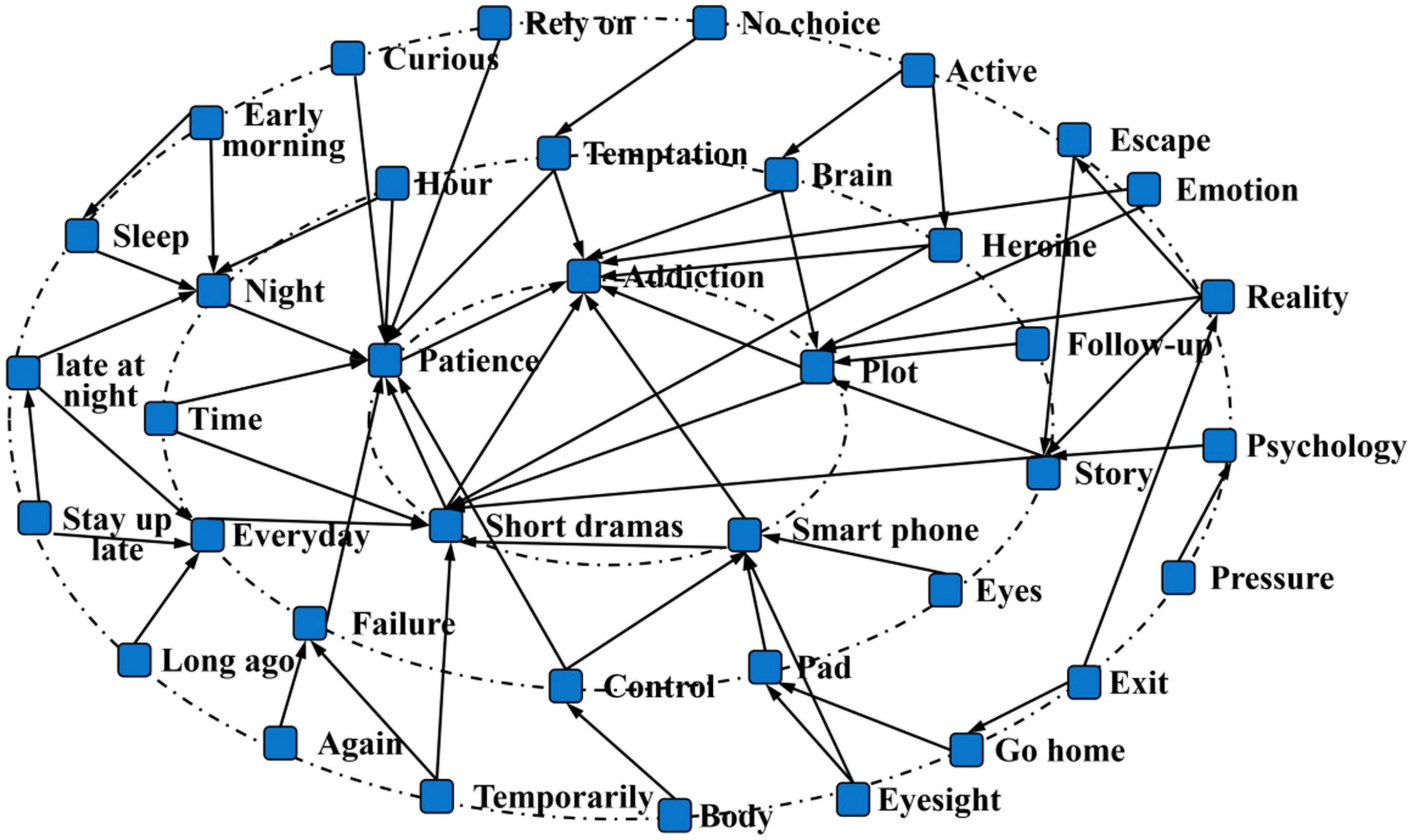
Personal mental map of participant 27.

The ZMET consists of 10 steps, which were strictly followed in this study:

- Step 1: Storytelling. Each participant was invited to explain the rationale and narrative behind each image they provided. This step enabled researchers to accurately grasp the core meaning of the images and clarify the underlying reasons for the participant’s image selection. Specific prompts included: (1) Please describe the content of this image; (2) Why did you select this image? (3) What specific thoughts and feelings about short drama addiction do you aim to express through this image? Follow-up questions were asked based on the participant’s narrative to deepen the inquiry.
- Step 2: Missing images. Participants were asked whether there were additional images that could convey their perspectives but were not included due to logistical constraints. If so, they were requested to describe the content of these absent images in detail. Researchers guided participants to elaborate on the feelings related with these images, to supplement the data collected in Step 1.
- Step 3: Image categorization. Without researcher intervention, participants were invited to subjectively categorize all provided images (including described missing images), explain the rationale for their categorization, and assign labels to each category. This step helped participants clarify their thinking and identify the relational links between images.
- Step 4: Construct extraction. Constructs were defined as words extracted from participants’ images and narratives that represented their views and experiences, and this step was the core of the ZMET. Researchers used the laddering method to guide participants to extract constructs from each image, using core prompts: (1) What is the relationship between this image and your views on short drama addiction? (2) What is the source of this relationship? Based on participants’ explanations, researchers identified new constructs and merged semantically synonymous terms. Laddering continued until no new constructs emerged. Based on the classification in step 3, taking participant 27 as an example, her construction extraction process is shown in Table 2.
- Step 5: Representative images. Participants were asked to select the most representative images most closely linked to the research topic, to reinforce the meaning of constructs and further expand their thinking.
- Step 6: Opposite images. Participants were invited to describe images that represent the opposite of their views on short drama addiction. This step enabled credibility assessment via negative examples, and expanded the depth of data via reverse thinking.
- Step 7: Multi-Sensory impressions. Participants were asked to describe their experiences of short drama addiction by using non-visual senses (hearing, touch, taste, etc.), to evoke metaphors beyond the visual medium of images.
- Step 8: Personal mental mapping. Each participant created a personal mental map based on the content of the previous steps, consisting of extracted constructs and the relational links between them. During this process, participants confirmed whether the constructs accurately reflected their thoughts and feelings, to validate the accuracy of construct extraction. Hand-drawn mental maps were standardized by using Rost 6.0 software, taking participant 27 as an example (Figure 1). The standardized maps were divided into three hierarchical layers: (1) the core layer (smallest circles), containing constructs representing the participant’s core views; (2) the secondary core layer (mid-sized circles), containing constructs linked to the core layer that provide supplementary explanation; (3) the outer layer (largest circles), containing constructs not directly linked to core views, derived from specific images or narratives but still reflecting the participant’s views. The links between constructs in each personal mental map represent the unique views of the individual participant, and all personal mental maps formed the foundation for the final consensus map.
- Step 9: Summary video. For each participant, interview data (provided images and interview text) were compiled into a summary video using video editing software, to present their views alongside the mental map. This step enabled participants to review their narratives via a multimedia medium, facilitating systematic verification and validity testing of the data. Images belonging to the same category were compiled using image processing software, imported into video editing software, and enhanced with introductory text, background music, narration, and transition effects to ensure clarity and accessibility. Participants were invited to review the video, with revisions made based on their feedback to ensure the content accurately reflected their views.
- Step 10: Consensus Map Construction. The final step of the ZMET was the construction of the group consensus map. Key constructs were extracted from participants’ personal mental maps, integrated into the consensus map, and linked based on their internal relational structure. Two convergence principles were followed for map construction: (1) included constructs must be mentioned by at least one-third of participants (*n* ≥ 12 in this study); (2) the link between any two constructs must be mentioned by at least one-quarter of participants (*n* ≥ 9 in this study; Christensen and Olson, 2002). The consensus map identifies core, academically relevant, and explanatory views related to the research question, providing an intuitive presentation of the collective consensus reached by participants on the topic. All constructs represented in the map were frequently referenced by participants during interviews, effectively capturing their feelings, attitudes along with the central issues and collective perspectives of the group (Zaltman, 1997).

**Table 2:** Step 3 and Step 4 results for participant 27.

Category number	Images’ names	Category	Representative concept
1	Image 1: Sofa and ipadImage 2: Emotional immersionImage 5: tangled phone charging cableImage 8: Power bankImage 9: Phone stand on the tableImage 12: Clock	Time management is out of control	sofa, tablet, staying up late, plot, bloodshot eyes, no drowsiness, household chores, mobile phone, playback, bowl, obsession, procrastination, scope, constraints, mobile phone charging cables, clutter, Anxiety, inability to stop, power bank, power supply, phone battery life, focus, inability to pause, phone holder, eating, watching while eating, distraction, dining table, killing time, resting, clock, time passing, delay, time management, loss of control
2	Image 7: Reciting linesImage 11: Screenshot of the CEOImage 10: Screenshot of the female lead in the short drama	Emotional immersion in short dramas	dialogue, speaking style, influence, dialogue immersion, life, family scenes, language imitation, handsome men and beautiful women, youth, nostalgia, screenshot, save, envy, longing, mobile album, plot fragments, character introduction, dream, comparison, mobile screenshot, mobile wallpaper, romance, dream attachment, emotional projection
3	Image 3: Presbyopia glasses Image 4: Short drama rechargeImage 6: Short drama push	Social loss	presbyopia glasses, vision, fatigue, non-stop, blurred, recharge, impulse, expensive, cost, trap, addiction, consumption, price, family, social, addiction, unread messages, temptation

## 3 Results

### 3.1 Consensus map

For the text data collected by ZMET, two trained researchers first coded the interview content, compiling all constructs from the personal mental profiles of 36 participants, yielding an initial set of 471 constructs. The reliability between encoders was tested using Cohen’s kappa coefficient, which was 0.83. This coefficient is above the acceptable threshold for inter-encoder reliability, indicating high consistency and reliability of the coding results. Following contextual analysis and semantic differentiation, semantically synonymous, abbreviated, or interchangeable constructs were merged to improve accuracy and validity, reducing the total to 158 unique constructs. In line with the pre-specified convergence principles, these 158 constructs were further refined by two researchers, resulting in a final set of 71 valid constructs (Table 3).

**Table 3:** Summary of shared constructs across all participants.

Serial number	Construct	Frequency	Serial number	Construct	Frequency
Original constructs: 18					
1	Decompress	36	10	Time	25
2	Convenient	34	11	Obsession	24
3	Boring	32	12	Fill	22
4	Easy	30	13	Young	21
5	Empathy	29	14	Close to life	20
6	Curious	28	15	Frequency	19
7	Attraction	28	16	Suitable	12
8	Stimulate	27	17	Lonely	12
9	Charm	26	18	Increase	22
Related constructs: 33					
1	Smart phone	36	18	Satisfaction	20
2	Binge-watch	34	19	Comfortable	20
3	Stay up late	32	20	From start to finish	19
4	Continuous	30	21	Tireless	19
5	Selection	29	22	Get up early	19
6	Habit	28	23	One by one	19
7	Repeat	27	24	Sober	18
8	Stop	27	25	Presbyopic glasses	17
9	Random	26	26	Cervical spondylosis	16
10	On time	26	27	iPad	15
11	Thinking	26	28	Sofa	14
12	Loop	26	29	Housework	13
13	Rewatch	25	30	Eat	12
14	Update	24	31	Sleep	12
15	Download	23	32	Advertisement	12
16	Dizzy	22	33	Television	12
17	Self-control	21			
Final constructs: 20					
1	Health	36	11	Vision	22
2	Pleasant	34	12	High-quality	21
3	Relaxing	32	13	Reasonable	20
4	Delay matters	30	14	Forgetfulness	19
5	Waste time	29	15	Quit an addiction	18
6	Sense of immersion	28	16	Enjoy	17
7	Distraction	27	17	Be ill	16
8	Emptiness	25	18	Protagonist	15
9	Physical and mental	24	19	Moderate	14
10	Restriction	23	20	Management	12

**Figure 2:**
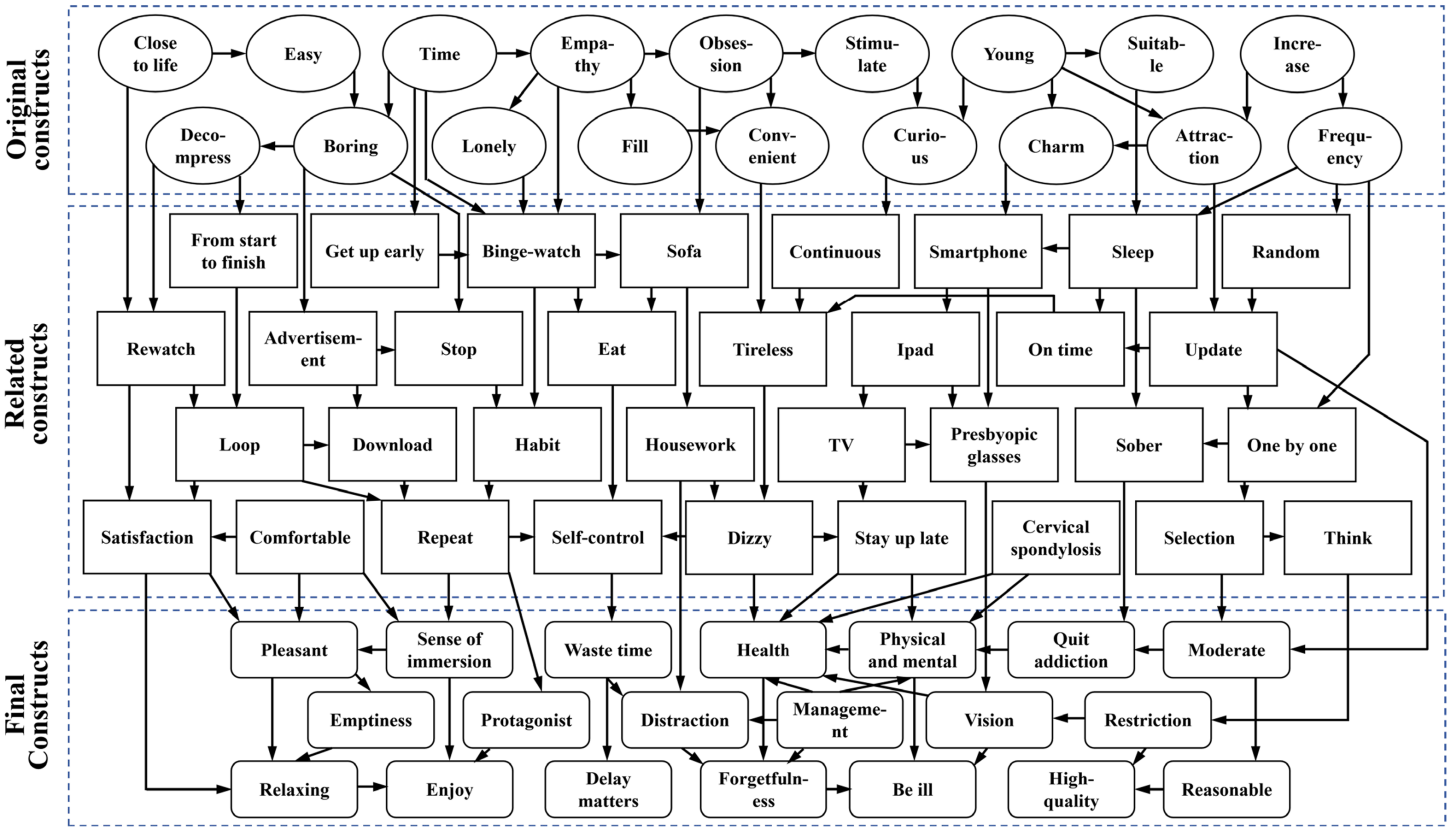
Consensus map of all participants.

These constructs were categorized into three types (Kong et al., 2018): (1) 18 original constructs, located at the base of the construct relational chain, representing the core triggers of participants’ experiences of short drama addiction; (2) 33 related constructs, located in the middle of the relational chain, acting as mediating links to describe the context, situation, or behavioral processes elicited by the original constructs; (3) 20 final constructs, located at the end of the relational chain, representing the ultimate outcomes of the linked constructs, as well as the final views of all participants on short drama addiction behavior. Finally, a group consensus map was constructed based on the relational links between constructs identified by participants (Figure 2).

### 3.2 Consensus of short drama addiction among Gen X women

Based on the consensus map, the relational links between constructs, and semantic logic, we identified four consensuses following the logical chain of “original construct triggering–associated construct mediation–final construct realization.” These four consensuses clearly outline the underlying antecedents, cognitive attitudes, awareness of harm, and intervention needs related to short drama addiction among Gen X women. This analysis reveals the internal psychological mechanisms and behavioral logic associated with this phenomenon.

#### 3.2.1 Consensus 1: Short dramas provide emotional value and psychological compensation

This consensus comprises six final constructs: “pleasant,” “relaxing,” “enjoy,” “sense of immersion,” “protagonist,” and emptiness. The original constructs, such as “decompress,” “empathy,” and “boring,” along with related constructs like “binge-watch,” “comfortable,” and “rewatch,” establish a framework characterized by the pathway of “psychological need triggering—media consumption behavior mediation—emotional value and psychological compensation acquisition.” This framework elucidates the fundamental logic and developmental process underlying short drama addiction among Gen X women.

The original construct “decompress” appeared 36 times throughout the entire sample, establishing it as the most significant triggering factor. This frequency directly supports the assertion that the primary motivation for Gen X women’s engagement with short dramas is the need for emotional release. Participant 29 stated: “I have to care for a sick elderly man every day and manage all the household chores. Sometimes I feel like I cannot breathe. Only when I watch short dramas can I temporarily let go of all the pressure, not think about anything, and feel so much more relaxed after watching.” This narrative directly illustrates the core connotation of the decompress construct. The original constructs “close to life” and “easy” were closely linked. Participant 17 noted: “The conflicts between middle-aged couples and the estrangement between parents and children depicted in short dramas are so similar to my own family situation. Watching the characters struggle and reconcile, I feel a deep sense of resonance, like someone finally understands my hardships.” As Gen X women navigating the dual responsibilities of family caregiving and career development, participants universally reported experiencing chronic emotional exhaustion. The low cognitive demand and minimal emotional labor required for short drama viewing enable Gen X women to achieve immediate stress relief and emotion regulation.

About related constructs, “binge-watch” was the primary behavioral pathway through which participants released emotions and obtained psychological compensation. Participant 27 mentioned: “Every day after work, I put aside my housework and immediately start watching short dramas. I watch for hours at a time. The plots are amazing, and I rewatch the scenes where the main characters resolve family conflicts over and over. The more I watch, the more comforted I feel, like my stress is melting away.” This narrative reflects the “binge-watch” construct, while echoing the original constructs of “decompress” and “boring.” Participant 15 stated: “Watching short dramas has become a habit for me. I watch a little when I wake up in the morning, a little during my lunch break, and even during breaks from housework. If I do not watch for a day, I feel like something is missing.” This statement clearly presents the “loop” and “satisfaction” constructs, reflecting that short dramas have become deeply integrated into participants’ daily lives as their primary means of alleviating boredom and filling emptiness. These related constructs transform the psychological needs in the original constructs into concrete behaviors, driving the formation of the final constructs.

About final constructs, “pleasant” was the most direct emotional descriptor of participants’ short drama viewing experience. Lighthearted plots and positive narratives rapidly alleviated their daily emotional stress, delivering immediate pleasure and serving as a core outlet for negative emotions. “Relaxing” was the primary demand driving Gen X women’s short drama viewing. Participants universally reported that short dramas enabled temporary physical and mental relaxation under the dual pressures of family and career, delivering psychological relief. Enjoy reflected the immersive experience of Gen X women during viewing, during which they could temporarily escape the trivialities and troubles of reality and immerse themselves in the plot. “Sense of immersion” was the core link between short drama content and participants’ psychological needs. Plots depicting middle-aged family dynamics and other relatable themes generated strong resonance with Gen X women’s real-life experiences, enabling them to project their emotions onto drama characters and obtain psychological comfort. The “protagonist” fulfilled this group’s psychological compensation needs: middle-aged Gen X women often occupy multiple supporting roles in real life, leading to their sense of self-worth being overlooked. By watching the protagonist’s narrative in dramas, they compensated for the lack of self-identity in reality. “Emptiness” was a key antecedent of short drama addiction; as their children leave home or careers plateau, Gen X women often experience a lack of meaning in life, and short dramas become a core vehicle for filling fragmented free time and alleviating loneliness, further strengthening their emotional dependence.

#### 3.2.2 Consensus 2: Short drama addiction represents a failure of attention and time management

This theme comprises five final constructs: “delay matters,” “waste time,” “distraction,” “forgetfulness,” and “management.” The original constructs of “time,” “convenient” and “fill,” along with related constructs such as “eat,” “self-control” and “housework,” collectively establish a comprehensive pathway: “filling fragmented time—reinforcing behavioral inertia—loss of attention and time management control.” It clearly elucidates the progression of Gen X women from casual leisure activities to addictive behaviors and a loss of time management control. Additionally, it presents the group’s self-attribution and reflective analysis regarding their addiction to short dramas.

About original constructs, “convenient” was mentioned 34 times, directly reflecting that the low barrier to access and high accessibility of short dramas are critical prerequisites for the loss of time management control. Participant 11 mentioned: “Short dramas can be watched instantly. While cooking or waiting for my kids to get home from school, I can easily watch a couple of episodes. I only intended to watch for a few minutes, but before I knew it, an hour or two had passed.” This statement describes the connotation of the “convenient” construct, while echoing the “time” construct. “Time” is linked to multiple original constructs and is the initial motivation for many of them. Participant 4 stated: “After my child went to university, the house suddenly felt empty. After retiring, I had nothing to do, and I did not know what to do with all my free time. Watching short dramas does not require much thought, and time just flies by.” The “fill” construct indicates that when Gen X women face a time void caused by shrinking social roles and a slower pace of life, short dramas become their preferred way to fill fragmented time. This initial “no-purpose, on-demand” viewing mode foreshadows the subsequent loss of time management control.

About related constructs, “self-control” confirms the core attribution of participants’ behavioral loss of control. Participant 23 mentioned: “I know I still have chores to do and meals to prepare, but I just cannot stop. I keep thinking I’ll do them after this episode, and then one episode after another, until I’ve finished the whole series. I completely lose control of myself.” This statement echoes the “control” and “rely on” constructs in her personal mental map, directly interpreting the connotation of the “self-control” construct. The “eat” and “housework” constructs clearly demonstrate the impact of short drama addiction on daily life scenarios, significantly encroaching on time originally allocated to household tasks and normal life routines. Participant 8 mentioned: “I used to get up in the morning to tidy the house, buy groceries, and cook, but now the first thing I do when I open my eyes is watch short dramas. Before I know it, it’s noon, and none of my housework is done; I even watch short dramas while eating. It feels like short dramas have become an indispensable part of my daily life.” These related constructs present the evolution of Gen X women’s short drama addiction from “fragmented entertainment” to “full-scene coverage,” and directly link to the final constructs.

About final constructs, “waste time” and “delay matters” are the most direct negative evaluations of short drama addiction from this group. Participant 31 said: “Looking back, I spent four or five hours a day watching short dramas, and got nothing productive done. I did not even finish my housework. So much time was wasted, and I regret it when I think about it.” This statement directly echoes the “time” original construct, forming a cycle of “starting to kill time, ending up wasting time.” “Distraction” reflects the profound damage short drama addiction causes to this group’s attentional capacity. Participant 14 mentioned: “Now I cannot concentrate on anything. When I’m cooking, I’m thinking about the plot; when I’m talking to someone, I’m wondering what happens next. I’m easily distracted and cannot focus on completing a single task.” “Forgetfulness” results directly from prolonged distraction. Many participants reported experiencing memory loss and difficulties in retaining information following extended engagement with short dramas. Common narratives included statements such as: “I was just about to get something, but the plot interrupted me, and I immediately forgot what I was supposed to do.” “Management” signifies the group’s profound contemplation of their behavior. Participants collectively acknowledged that addiction to short drama is fundamentally a failure in both time management and attention management. This recognition establishes a basis for the subsequent demands for behavioral correction.

#### 3.2.3 Consensus 3: Short drama addiction exerts negative impacts on physical and mental health

This consensus comprises four final constructs: “health,” “physical and mental,” “vision,” and “be ill.” Together with original constructs including “obsession,” “stimulate,” and “charm,” and related constructs including “stay up late,” “presbyopic glasses,” and “cervical spondylosis,” it forms a pathway of “immersive viewing–continuous physiological depletion–irreversible physical and mental damage.” The pathway presents the evolutionary process from physiological discomfort to health damage experienced by Gen X women, while revealing this group’s awareness of the physiological harms of addictive behavior.

About original constructs, “stimulate” mentioned 27 times, is a significant factor influencing immersive viewing and contributing to both physical and mental harm. This finding directly supports the notion that the highly conflict-driven content of short dramas serves as a primary catalyst for excessive viewing. Participant 16 mentioned: “Every episode of a short drama has a conflict. As soon as I finish one episode, I want to know what happens in the next. The plot is so exciting that I cannot stop watching. I often watch for most of the day, without even having time to drink water.” The “obsession” and “charm” constructs further drive sustained viewing. Participant 22 stated: “The characters and plot of the short dramas are so captivating; I’m completely drawn in. Sometimes, to catch up on new episodes, I set an alarm to get up in the middle of the night to watch, even knowing that staying up late is bad for my health. I just cannot resist.” This unrestrained, high-intensity viewing behavior driven by these original constructs directly sows the seeds for subsequent physical and mental harm, becoming the starting point for addictive behavior and health hazards.

About related constructs, “stay up late” became the mediating behavior that triggered physical and mental harm, and is also the most typical behavioral manifestation of short drama addiction among Gen X women. Participant 9 mentioned: “Every night I lie in bed and start watching short dramas. I intend to watch two episodes and then go to sleep, but the more I watch, the more awake I become. By the time I realize it, it’s already 3 or 4 in the morning. The next day, I wake up with a headache and feel unwell all over.” The “presbyopic glasses” and “cervical spondylosis” constructs explain the physical discomfort caused by prolonged short drama addiction. Participant 25 stated: “My eyesight is already blurry, and looking at my phone for a long time makes it even worse. Now I have to wear reading glasses to watch short dramas. After watching, my eyes are so dry I cannot open them, my neck is stiff and I cannot move, and my head is dizzy. It takes a long time to recover.” These constructs describe a vicious cycle of bad habits and negative consequences, ultimately leading to various health problems.

About final constructs, “health” confirms that participants are aware of the negative impact of short drama addiction on their physical health. Participant 33 mentioned: “My health is getting worse and worse. My blood pressure is high, and I do not sleep well. The doctor said it’s because of watching too many short dramas. It’s not worth ruining my health for a few short dramas.” This statement directly links short drama addiction to adverse health outcomes. The “physical and mental” construct indicates that Gen X women have broadened their understanding of the harms associated with short drama addiction, encompassing both physiological and psychological dimensions. Many participants noted that prolonged engagement with short dramas not only resulted in physical discomfort but also precipitated psychological issues such as low mood, lethargy, anxiety, and irritability. “Vision” was the most frequently mentioned specific physical damage; as a direct consequence of prolonged close-range electronic screen viewing, vision decline and related problems are prevalent among participants. Participant 7 stated: “I used to have very clear vision, but in the two years I’ve been watching short dramas, my eyesight has deteriorated rapidly. Now even distant objects are blurry. The doctor said it’s from excessive eye strain and told me to reduce my phone use, but I just cannot quit.” “Be ill” highlights the serious consequences of addictive behavior. Some participants indicated that extended and unrestrained viewing has resulted in various chronic illnesses, thereby enhancing this group’s comprehension of the detrimental effects associated with short drama addiction. These descriptions of “vision” and “be ill” are subjective accounts from the participants. Although there is no medical clinical evidence that “short drama addiction” is the cause of these illnesses, the participants’ statements demonstrate their concern for health.

#### 3.2.4 Consensus 4: Participants demonstrate awakened awareness and attempts to correct addictive behaviors

This consensus comprises five final constructs: “quit addiction,” “restriction,” “moderate,” “reasonable,” and “high-quality.” Together with original constructs including “frequency,” “attraction,” and “suitable,” and related constructs including “selection,” “think,” and “sober,” it forms a transformation pathway of “harm awareness–healthy consumption demands–behavioral correction attempts.” This presents the psychological shift of Gen X women after experiencing the negative consequences of addictive behavior, from passive indulgence to active intervention, and from unrestrained consumption to rational demands.

About original constructs, “frequency” directly reflects participants’ cognitive shift from “unrestrained indulgence” to “active control of viewing frequency.” Participant 21 mentioned: “I used to watch whenever I wanted and as long as I wanted, but now I realize that I cannot continue like this. I have started to consciously control the frequency of watching short dramas, only watching them for a short time after finishing housework at night.” This statement indicates that this group has developed self-control awareness regarding addictive behavior. The construct “suitable” encapsulates this group’s definition of healthy viewing behavior. Some participants contend that short dramas do not represent a wholly negative form of entertainment. They emphasize the importance of identifying an appropriate viewing time and context to strike a balance between entertainment and daily life, thereby fostering a more rational understanding. “Attraction” reflects the contradictions in this group’s cognitive awakening process, even after recognizing the dangers of addiction, short drama content remains highly appealing. Participant 35 stated: “I know I should not get addicted, but sometimes when a new drama is released, the plot is still very attractive, and I cannot help but want to watch it. I can only repeatedly remind myself to restrain myself.” These constructs demonstrate the most typical psychological characteristics of this group’s cognitive awakening stage, and lay the foundation for subsequent behavioral modification attempts.

About related constructs, “think” and “sober” are important constructs linking cognitive awakening and behavioral modification, together forming the psychological basis for this group’s shift from passive addiction to active intervention. Participant 30 stated: “In the past, when I watched short dramas, I was completely led by the plot. Now, I consciously think about what I can get from watching this drama, whether it’s worth spending so much time, and whether it will delay important things for me. Once you think it through, you can better control yourself.” This statement reflects the group’s rational understanding of short dramas. “Selection” reflects the group’s behavioral correction in practice, participants have shifted from passively receiving information to actively choosing content. Participant 13 mentioned: “I used to watch any drama I came across, and the more I watched, the more addicted I became. Now, I read the synopsis first, only choosing high-quality short dramas, and directly skipping low-quality ones.” These related constructs present the group’s complete process from cognitive awakening to concrete behavioral correction, and directly contributed to the formation of the final constructs.

About final constructs, “restriction” is the primary behavioral correction practice for Gen X women, reflecting participants’ most direct intervention in addictive behaviors. “Quit addiction” reflects participants’ strong desire to completely break free from addictive behavior. Many participants reported trying strategies including uninstalling short drama apps and enlisting family supervision, and despite relapses, continue to make attempts. The “moderate” and “reasonable” constructs reflect a more mature and rational media consumption view among this group. Participants universally believe there is no need to completely deny the entertainment value of short dramas. The primary objective is to attain “moderate viewing and reasonable consumption,” positioning short dramas as a means of regulating emotions and enhancing life, rather than as an addictive force that overshadows daily existence. “High-quality” reflects this group’s positive demands for short drama content. Over two-thirds of participants expressed a desire for short drama platforms to introduce more high-quality productions that resonate with the experiences of middle-aged women, feature uplifting themes, and exhibit high production standards. This demand not only provides external support for the behavioral correction of Gen X women, but also provides a clear user orientation for the content optimization of the short drama industry.

## 4 Discussion

### 4.1 Key findings

This study employed ZMET combined with semi-structured in-depth interviews to explore problematic short-drama addition among Chinese Gen X women. Through hierarchical construct extraction and personal mental mapping, 71 constructs were obtained, and a consensus map of the participants was created. Rather than “fully reconstructing” a definitive developmental process, the findings provide an exploratory qualitative account of how participants themselves perceived the psychological meanings, behavioral experiences, and self-regulatory challenges associated with intensive short-drama viewing. The study confirms that long-term emotional exhaustion under the dual pressures of family and career is the core factor in this group’s addictive behavior. “Decompress” was the only construct mentioned by all participants. The low cognitive threshold and strong emotional feedback of short-dramas help them achieve immediate stress release. The emotional empathy brought by realistic narratives and the self-worth compensation provided by the protagonist’s narrative further strengthen the group’s emotional dependence. The high convenience and strong plot of short dramas have gradually led to the depletion of participants’ self-control resources, causing a double loss of control over time and attention management. This is the key intermediary link in the development of this group from daily leisure viewing to problem addiction.

Research has found that short dramas addiction has a dual negative impact on Generation X women, both physiologically and psychologically. Prolonged immersive viewing not only causes widespread physical symptoms such as vision impairment, and sleep disorders within the group, but also exacerbates their psychological emptiness and sense of social isolation. Furthermore, participants have developed a comprehensive and clear understanding of the harms of addictive behavior. Unlike traditional research conclusions that addicts perceive low harm, participants in this study did not completely deny the entertainment value of short dramas. Instead, they formed a rational media consumption view centered on “moderate viewing and reasonable consumption.” They have attempted to correct their addictive behavior by controlling viewing frequency, defining viewing scenarios, and actively selecting high-quality content. Simultaneously, they showed a clear demand for high-quality short dramas that resonate with the lives of middle-aged women. This study breaks through the paradigm limitations of previous studies that focused on adolescents and emphasized the identification of explicit risk factors. It reveals the social structural motivations and profound psychological rationale for addictive behavior in middle-aged women, addressing a theoretical void in media addiction research concerning distinct generations and gender groups. Additionally, it offers robust empirical evidence to steer healthy digital consumption practices and develop tailored addiction intervention approaches.

### 4.2 Comparison with previous studies

While no previous research has specifically focused on short drama addiction among Gen X women, the findings of this study align with several existing studies on internet addiction and short video addiction (Ye et al., 2022; Ye et al., 2024). Regarding the developmental pathway of addiction, the low threshold of short dramas positions them as a primary choice for occupying fragmented time. As self-control resources become depleted, the management of time and attention can spiral out of control. This observation aligns with the perspectives of researchers examining micro-short dramas within the new media environment (Liu and Bexci, 2025). The negative consequences of short drama addiction encompass both physical and psychological harms. Prolonged viewing can result in vision and cervical spine issues, while also intensifying feelings of psychological emptiness and social isolation. This observation is consistent with the analysis by Dong et al. (2024), which examines the impact of short video content characteristics on addiction to short video apps and its underlying. This study confirms that the needs for emotion regulation and low self-control are significant factors contributing to short drama addiction among Gen X women. These findings align with the research conducted by Ye et al. (2025) on short video addiction.

The key differences between this study and existing studies lie in the conceptual paradigm and research perspective. Previous studies have defined addiction as an individual’s cognitive and behavioral biases, with a focus on analyzing the negative effects of risk factors (Brewster and Padavic, 2000). In contrast, this study contextualizes the short drama addiction of Gen X women within the framework of evolving gender roles and social transformation. It interprets the psychological compensation needs and role conflicts underlying this behavior and delineates a dynamic process characterized by “trigger—development—awakening.” This approach aligns with the research of Shuja and Wazir’s (2025) focusing on the cognitive consequences of media addiction, while enriching the research perspective of media addiction.

Current research on group heterogeneity and methodology primarily examines adolescents and middle school students. Scholars widely concur that this demographic exhibits a lower resistance to internet temptations compared to adults (Lu et al., 2022). This study focuses on Gen X women as the research subject. Beyond the differences in the study population, the emotional pressure experienced by Gen X women primarily arises from the dual burden of family responsibilities and career development. This distinction underscores the intergenerational heterogeneity of the factors driving media addiction. In terms of methodology, current research on media addiction is predominantly quantitative (Yao et al., 2025). Questionnaire surveys and structural equation modeling have long been the dominant research methods in the fields of short video addiction and internet addiction. This study innovatively adopts the ZMET to address the limitations of quantitative research, uncover hidden constructs, supplement research on the deep psychological mechanisms of media addiction, and complement existing research methodologies.

### 4.3 Limitations and future directions

This study based on the ZMET method, reveals the psychological mechanisms and behavioral logic of short drama addiction among Chinese Gen X women. However, several limitations should be noted, which provide avenues for future research. First, this study adopted a cross-sectional qualitative design, which cannot establish causal relationships or dynamic evolutionary trajectories between addictive behaviors and their influencing factors. Second, snowball sampling limited the demographic diversity of the sample, and the generalizability of the findings requires further verification. Third, the data relied on participants’ self-reports and interview narratives, which may be susceptible to social desirability and recall biases. Moreover, the lack of cross-validation with objective behavioral data means the robustness of the findings can be further improved. Finally, the study did not incorporate external variables such as family support and platform algorithms, resulting in insufficient exploration of intra-group heterogeneity.

Future research could address these limitations via the following avenues: (1) conducting longitudinal studies to clarify the developmental trajectory and causal mechanisms of short drama addiction; (2) employing mixed-methods designs to validate the core conclusions of this study and construct a more explanatory integrated theoretical model; (3) expanding sample coverage to explore intra-group heterogeneity and intergenerational differences in short drama addiction; (4) examining the synergistic effects of multi-dimensional factors to develop targeted and effective intervention programs, providing further empirical support for short drama addiction research.

## Data Availability

The raw data supporting the conclusions of this article will be made available by the authors, without undue reservation.
